# Book Reviews

**Published:** 1828-01

**Authors:** 


### CRITICAL ANALYSES.

Quæ laudanda forent, et quæ culpanda, vicissim

Illa, prius, cretâ; mox hæc, carbone, *notamus.*—Persius.

*An Introduction to the Comparative Anatomy of Animals; compiled with constant reference to Physiology, and illucidated by twenty Copper-plates.* By C. G. Carus, Med. et Phil. Doct. Professor of Midwifery to the Medico-Chirurgical Academy at Dresden; Director of the Royal Saxon Obstetrical Institution at the same place; and Associate of various learned Societies. Translated from the German, by R. T. Gore, Member of the Royal College of Surgeons in London. In two Volumes.—8vo. Longman and Co. London, 1827.

It is not our usual custom to analyse works of an elementary nature, such as the one before us; but when we consider how much less the study of comparative anatomy occupies the attention of the student in this country than it ought to do, we think we shall confer a favor on our readers by laying before them a sketch of the work of Carus, such as we hope will induce them to turn to the original for further information.

The structures first considered are those belonging to animal life: the Zoophyta, as lowest in the scale, are those with which the volume commences. The author combats the idea that the different elementary functions must necessarily be distributed among distinct structures. When, says he, we find that there may be respiration without lungs,—nutrition, growth, and secretion without a circulation of fluids,—generation without distinct sexes, why should we doubt that sensitive life may exist without nerves, or motion without muscular fibres?

The investigation of the anatomical composition of zoophytes has not hitherto discovered, in the lower orders of creation, any thing more than an uniform gelatinous mass; and yet when we observe that the sensibility of these animals, simple in construction as they are, is of great acuteness,—that some of the marine polypi have been ascertained always to turn towards the light, while others display an aversion to it;—when also we perceive that the lower orders of zoophytes possess the power of motion in a high degree,—we cannot, according to M. Carus, hesitate in considering this uniform gelatinous mass as the primitive animal structure, which, in the same manner as it furnishes the groundwork for more perfect textures in the embryo of the higher classes of animals, is here destined for the exercise not only of the various animal, but likewise of the vegetable functions. This view would enable us to explain the extraordinary power of reproduction enjoyed by these animals, by means of which parts are so readily replaced, or even converted into new beings.

The uniformity of structure, however, in these animals leaves but little room for anatomical description. Even locomotion, so characteristic of volition, seems, in many of these animals, to be of a passive nature. The Medusæ, being nearly of the same specific gravity as the water they inhabit, are moved by the waves and currents; while some (the Holothuria physalis) appear to sail. Some are capable of performing a progressive motion by fixing the head and tail alternately; others present the appearance of attraction and repulsion, as in electrical action, and yet others (corals and sponges) are wholly incapable of locomotion.

In the movements proper to the body, there is a remarkable coincidence with those of plants; the arms of the polypes retracting towards the centre when stimulated, as the stamina of some flowers towards the stigma; and the polypiform organs, called by our author the "animal blossoms," of the Gorgoniæ, and others, fold themselves up, just in the same manner as the leaves of the Mimosa.

The Infusoria are little more than cells partially filled with lymph, and the innumerable variations of form they assume may easily be accounted for by the different degrees and extent to which the fluid is collected at one or other point of their bodies. In the polypes the arms are entirely composed of tubes communicating with the body, and containing fluid. The body contracting, forces the fluid into them, producing their elongation; while the tube contracting, returns the fluid, and the arm is again retracted.

The organs destined for the sensitive functions seem to be the arms, and other projections with which they are furnished. These organs, however, serve other purposes, particularly prehension and motion.

In those animals of this class which are possessed of a skeleton, it either forms an insensible stalk around which the elementary animal substance is fixed, or the latter is included within the former: with respect, however, to the solid parts, they differ essentially from bone, being either of a horny texture, or almost wholly composed of carbonate of lime.

There are some other genera of zoophytes, which, by the greater perfection of their organization, appear to form a transition to the other classes of animals. In these, nervous and muscular fibres are discernible. The nervous matter first presents itself as a *peripheral* layer round the common central cavity, which is at once a stomach, heart, and sexual organ. From this central mass nervous filaments are sent to supply the different parts, and the organs of motion are, under such circumstances, more perfectly developed.

The organs of the animal functions in the Mollusca and Articulata next come under consideration. The distinction between the fibrous and ganglionic portions of the nervous system is particularly well marked in animals possessing neither brain nor spinal marrow; and the ganglia in some of them are further remarkable for their colour. Thus, in the Helix *stagnalis* and *cornea* they are bright red; in the Aphysiæ, blackish red; and in the common fresh-water Muscle, they are bright yellow. As to the form of the nervous system, the circle round the œsophagus, which appeared in the preceding class, constitutes in these two its most uniform and essential part. This nervous ring either receives additions to its bulk by the formation of larger ganglia in its substance, which gradually approximate at the upper part of the animal, or the nervous rings are multiplied, surrounding the alimentary canal.

In some of the Mollusca, the form of the nervous system approximates to the type of the higher classes of animals; and in the Cephalopoda we find the rudiments of a true skeleton, though as yet only in the shape of cartilage. The purpose, however, of protecting the more important parts is distinctly attempted. The simplest form presents itself as a cartilaginous ring, which receives the nervous circle and the cerebral ganglion in a grooved depression on its internal surface, and at the same time permits the passage of the œsophagus through its centre: the cartilage, too, is perforated in various parts for the transmission of nerves. In the common Sepia (*officinalis*), under the cartilaginous ring there is also a dorsal plate of the same material, placed anteriorly, from which the arms proceed. This, however, as well as the dorsal bone (*os sepium*), is more immediately connected with the organs of motion than those of sensation.

Of the Articulata, we find that among some of the vermes (as intestinal worms, hydatids, and tæniæ,) the transition to the class of zoophytes is so complete, that no nervous system, distinct from the mass of the body, can be traced. In others, however, (as the leech and the common earth-worm,) a chain of ganglia is very distinct: in the latter, the nervous cord approximates to the form of the spinal marrow in the higher orders of animals.

In the Crustacea, another step is gained in the more perfect formation of the individual ganglia, and in the appearance of proper nerves of sense.

In Insects, infinitely diversified as they are, the ganglionic chain still remains the most important part of the nervous system: the ganglia, however, are fewer in number, and proportionably larger; and it is a most curious fact, that the more elevated organization of this kind generally presents only as the ultimate result of various metamorphoses, so that the larvæ have a nervous system corresponding to that of the vermes, while, in the fully developed insect, it is more perfectly formed.

*Organs of Sense.*

When the nervous system is once decidedly distinguished from the general mass of the body, its relation to the external world becomes mediate: it is influenced by impressions through the intervention of the organs of sense, and acts upon the external world by means of its organs of motion. To complete this connexion with external objects, different senses are required, but these are only developed gradually, and from the first and most simple sense,—viz. that of feeling. There are two principal surfaces, a dermoid and an intestinal, and it is inferred that there are two corresponding kinds of feeling. Each of these constitutes, as it were, the primordium whence more perfect senses are ultimately developed: thus, from the intestinal sense is derived the sense of taste, by the addition of peculiar organs at the cephalic extremity of the intestine; while from the dermoid sense is derived that of touch. A yet higher order of senses are those destined "for the reception of the most important and most general relations of the individual organism." Sight and hearing are here alluded to as conveying to the mind ideas of totality and individuality.

The remarks of our author are here confined to the organ of touch, smell, sight, and hearing, inasmuch as he looks upon the consideration of taste and sexual feeling as inseparable from that of the digestive and sexual organs in general.

Touch and smell are ranked together under the head of dermoid sense. In the Mollusca these senses are but imperfectly developed; nor are they in a state of much perfection in the Articulata. The antennæ of insects seem to be frequently organs of smell as well as of touch.

Hearing may be divided into that extent of sensation which merely judges between differences in the strength of sound, and that which appreciates its quality; and it is probable that in most invertebral animals there is either no sense of hearing at all, or only one by which the strength of the vibrations propagating the sound can be perceived. Of the Mollusca, distinct organs of hearing have been found in the Cephalopoda alone. We mentioned that in these the first rudiments of a skeleton appeared. In the anterior part of their ring-shaped cartilage is a tubercle without any external aperture, but having depressions for two small membranous bags placed close together, and on which the auditory nerves are distributed: each bag contains a portion of fluid and a solid body. This appears to be the simplest form of the labyrinth, which, in its full development in the human species, constitutes the most important part of the organ of hearing.

In the Crustacea, the organs of hearing can be traced; but in many insects which hear most acutely, and which themselves emit sounds, (as the grasshopper,) no such organs have been discovered.

The first appearance of sight is observed in the Gasteropoda, where the function is combined with that of touch, as in some genera (Clio, Scyllæa, and Lernæa,) there are two little black points, sometimes situated at the extremity, sometimes at the middle, and sometimes at the base of the tentacula, presenting internally the most essential parts of an eye. In some these minute eyes are placed at the point of the feelers, and admit of being retracted and protruded according to circumstances. In the Cephalopoda, the eyes are of more considerable size, and have muscles attached to them: they want both the cornea and aqueous humor, but the vitreous humor is present, and the structure of the eye must be regarded as well developed. In the Articulata, the eye is less perfect.

In concluding this part of the subject, the author makes the following remarks:

"So far of the organs of sense of invertebral animals, the review of which unavoidably suggests various questions to the observer of nature. For example: Do these animals, like man in his ordinary state, feel by individual organs?—are they not rather, on account of their less complete individuality, as displayed in the want of centrality in their nervous system, to be viewed as integral parts of the system of the universe?—are they not, for the same reason, like somnambulists, organs of sense throughout?—must they not, on the same account, receive impressions from the slightest changes in the external world, although, from the deficiency of distinct consciousness, their sensibility is so imperfect that they are unable clearly to perceive or estimate them?—and lastly, is it possible, without admitting such ideas, to give any satisfactory explanation of their prescience of weather, of their wonderful instincts, of the habits of insects, and of all those singular phenomena which excite the curiosity and astonishment of all who witness them? It is to be wished that general physiology may yet, if possible, solve these inquiries." (Vol. i. p. 89.)

*Organs of Motion.*

When solid bony structures are met with in the animal frame, a close connexion always exists between them and the organs of motion, although they are more immediately destined to protect the important parts of the nervous system. In the Mollusca, the muscles constituting the active organs of motion have uniformly a gelatinous appearance, and are remarkably distinguished from the firmer muscular fibres of the superior animals. In the Acephala, which have shells, these are moved by powerful muscles and ligaments, distinct from the muscular organs of locomotion, the motions of the shell being connected with respiration rather than change of place. The most remarkable organ connected with the muscular system is an expansion which surrounds the whole body: in some this assumes a more perfect development, and is called the cloak. The opening of the shell is effected by a fibrous ligament at the hinge, the elasticity of which makes them gape; and, as an antagonist to this, there is generally a short strong muscle at the anterior and posterior parts of the shell, attached to both of them, and which serves to bring them together. In several of the Acephala, there is an organ called the foot, which effects locomotion and various other purposes. It is a fleshy mass, which can be projected and retracted, and which in some genera (Mytilus, Pinna, Aurcula,) has at its base the excretory duct of a gland, pouring out a tenaceous mucus, which the animal draws into threads, and thus attaches itself to the rocks.

In the Gasteropoda, which have no shells, the most important muscular organ is an external membrane, forming a bed in which the viscera are contained. Strong fibres cross each other in all directions, particularly over the abdomen, on which the animal crawls; and hence the name (Gasteropoda) given to this order. Mucus is copiously secreted from this part, by means of which the animal fixes itself, as well as by attaching the edges, and then retracting the central parts, so as to produce a tendency to a vacuum.

In Snails with shells there is a similar mechanism, but with a fissure on the dorsal side of the membrane, through which nearly all the viscera protrude (as in a hernial sac) into the shell. The foot is attached by muscles to the shell, by which the animal can retract itself within it. The forms of the shell in the Gasteropoda are exceedingly various, but it is remarkable that they almost all observe a uniformity with respect to the spiral turns, which are scarcely ever to the left side.

In the Cephalopoda there is a fleshy coat, but it appears to serve chiefly for the purposes of respiration, while the arms fixed round the head are the organs of locomotion. These arms are very various in shape and number; they are fixed round the head, (hence the name Cephalopoda,) are provided with suckers, and have a remarkable power of reproduction. In the Articulata there is no trace of any internal solid support to the moving powers, though there is occasionally an external case, or shell; and in some instances this is articulated, forming, as it were, an external moveable skeleton. The muscular fibres are more developed in this than in the preceding class: indeed, the muscular power in insects is very great in proportion to the size of the body,—greater than in any other instance; a circumstance connected with the perfection of the respiratory function in these animals. Locomotion is performed in the Crustacea by means of tolerably firm muscles, which are placed within the bony cylinders constituting the skeleton, which, as in the crab, for example, is external. The entire leg in these presents a prototype of the leg of the quadruped, the situation of the hard and soft parts being reversed. A remarkable circumstance connected with these is the facility with which these limbs are detached: thus, it is said that the lobster, when alarmed, can detach them by a voluntary effort.

The structure of the legs in Insects corresponds pretty closely with that of crabs, each phalanx forming a hollow tube, which contains the one beyond it. The wings of insects deserve particular attention. According to Oken, they are "detached bivalve shells, and hence their position on the back: the elytra represent the shells, and under them are the laminæ of the gills; the wing coverings are opercula." The wings are attached to the pectus, and moved by muscles placed within it, the veins of the wings apparently serving the office of tendons. It has already been mentioned, in speaking of the nervous system in insects, that, during their larva state, this descended in the scale approximating to the formation of the vermes. The same holds good with respect to their organs of locomotion. They have generally a layer of muscular fibres under the external covering; and to such extraordinary minuteness has the investigation of these objects been carried by some, that LYONNET has reckoned 4061 muscles in the caterpillar found on the willow.

*Second Formation of Organs belonging to the Animal Sphere.*

This part of the work describes the further development of the nervous, sensorial, and locomotive systems in animals with vertebræ; and, as the osseous frame is equally connected both with the nervous and muscular functions, so the skeleton now comes to occupy a principal share of attention, and its peculiarities are described in Fishes, the Amphibia, Birds, and Mammalia. Into this part of the work it is impossible for us to follow our author, and we must content ourselves with a few very general observations.

In all animals having a brain and spinal marrow, the fundamental form of the osseous frame is originally determined by the peculiar form of the nervous system. This consists of a single central mass extended along the back, and composed of a series of distinct portions, each of which, like the cerebral ganglions of one of the Sepiæ, is indicated by its giving off nerves; and it is a striking coincidence with such a disposition, that the principal portion of the skeleton is formed of a series of bony rings, which, being articulated so as to form a closed cavity, compose the cranial and vertebral column, the distinctive character of the second division of the animal kingdom. As the central nervous mass is separable into two great divisions, the brain and spinal cord, the former being distinguished by a higher degree of development; so also in the vertebral column, we may distinguish between the cranium and the spine; the former being, as it were, the ultimate development of the latter.

It is the object of M. Carus, in the descriptions which follow, to elucidate, by a series of animal forms, the nature and manner of this development,—the gradually increasing distinctness in the separation of the cranium from the spine, and the progressive advances in the formation of extremities, —to show, in a word, how, "at every step we take, the invariable uniformity of the productions of nature to fixed laws is evinced by proofs of every description."

With regard to the formation of bone, it is remarkable that, as the first indication of an internal skeleton consist of cartilage, (the cartilaginous ring of the Sepiæ, already described,) so also, in the higher animals, the rudiments of the skeleton is composed of the same material, and, while bone is formed by depositions of successive layers from secreting surfaces, so the earthy shells of the Molluscæ are formed by secreting membranes; with this difference, however, that in the higher animals the bone consists of phosphate of lime, and continues to be nourished, whereas in the other case the shell is composed of carbonate of lime, and is not changed after its first formation.

Referring to the work itself for a minute account of the skeletons of the various tribes of animals, we now resume our sketch of the volume, at that part where M. Carus proceeds to trace the development of the nervous system in the higher classes of animals.

Turning for a moment to the forms of organization of which we have already spoken, it will be remembered that the Articulata have, in accordance with the jointed structure of the whole body, an articulated nervous system—the ganglionic chain. The articulation of the body, i. e. its division into segments, still continues in the higher classes, where it is most distinctly marked in the spinal column. In the vertebrated animals, the central masses are consolidated so as to form a single and continuous cord; a change, however, of which we have some examples in the Vermes, Crustacea, and larvæ of insects. It is owing to this consolidation that the individual sections of the ganglionic chains in the spinal marrow are only to be distinguished by the pairs of nerves which they send off; but, when a more perfect evolution of the individual portions take place, as in the brain, the evidence of the existence of separate masses is no longer doubtful. From the brain uniformly proceed the nerves of hearing, sight, and smell, and consequently the number of its principal divisions is always fixed at three. Thus, one principal division may be proved as the ganglion of the olfactory nerve; the second, as the ganglion of the optic nerve; the third, as the ganglion of the nerves of hearing, smell, touch, and motion, (the latter two through the medium of the spinal marrow.) But in the higher classes of animals there is a peculiar nervous system for the vegetative functions, and which forms a perfect repetition of the nervous system of the inferior classes, both by surrounding the alimentary canal and vascular system, and also by presenting the type of a chain of ganglia.

In fishes, the brain and spinal cord maintain nearly equal rank; for, although the former has the preponderance in point of structure, yet the latter always has the superiority as regards bulk: indeed, it stretches through the whole extent of the vertebral column, even through the caudal vertebræ, which is not the case in the superior animals. The form of the spinal cord very closely resembles that of man: its nerves rise by superior and inferior roots, and it is only on the latter that ganglia are found. The brain in these animals is little else than a series of pairs of ganglia, ranged on the upper side of the medullary cord: its size is very inconsiderable. In some species of shark, the anterior mass consists of a single large ganglion, whence proceed the olfactory nerves, and which is remarkable as containing a cavity corresponding to the two lateral ventricles of the human brain; and these are invariably present in the succeeding classes. With few exceptions, the optic nerves cross without decussating, but they are connected by a commissure at their origin. In the third cerebral mass, there is a single ganglion, principally composed of grey substance, which may be considered as the prototype of the cerebellum; and, in the cartilaginous fishes, it corresponds in structure to that of the human subject.

In the Amphibia, the great divisions of the nervous mass are still nearly similar; for, although the brain is more developed than in fishes, yet the spinal marrow preponderates in point of size. The nerves are essentially distributed as they are in man; those only being absent which correspond to organs which do not exist.

In Birds with organs, viz. the brain and spinal marrow are developed in a higher degree than in any of the preceding. The brain has acquired a greater breadth and more rounded form, and now preponderates in point of bulk. The three primary divisions of the brain are no longer placed one behind the other, as in the preceding classes, but seem rather situated below each other. The optic nerves are generally very large; in this respect only to be equalled by those of the lizard, and form a perfect decussation.

In the Mammalia, the spinal marrow no longer admits of comparison to the brain with regard to size; nor to the same part in birds, as respects the perfect development of particular parts: in fact, the spinal marrow now becomes gradually more and more subordinate to the brain. The spinal cord, too, is in general much more similar to that of man than in the preceding classes. Of the individual masses composing the cerebrum, we may mention that the corpus callosum and fornix now present themselves in a well-developed condition, and the cerebellum receives a notable increase of size, and the spinal and cerebral nerves have a distribution in all essential circumstances similar to that in man.

One great object of our author throughout is to establish what he calls the "absolute centricity" of the nervous system in man; and he concludes this division of his work with the following pertinent remarks:

"In my Essay on the Nervous System, I have already claimed attention to the evident appearance of this principle of centricity in the history of the formation of the nervous system; nor will any unprejudiced person refuse to admit the manifestation of that law, if he carefully compare the various forms of that system, from the circular collar around the œsophagus in the lowest classes up to the human brain, preponderating alike over nerves and spinal marrow. This is not the place to examine the advantages accruing to physiology from the knowledge of that law; it appears, however, worth while to inquire whether the investigation of the nature of the mental faculties may not be promoted at least as much by such anatomical comparisons as by the performance of rude experiments on the brain of living animals? and which process of the two is really most suited to the nature of the object?

"The opinion advanced in that Essay, that the contemplation of the nervous system, as recognised by our external senses, is, as it were, the assignment of a locality in the organism for the intellectual faculties; and, on the contrary, the mind, as recognised by our internal senses, the nervous action viewing itself intuitively:—that opinion, I say, will not be refuted by quoting, in opposition to it, the anthropomorphous form of the brain of apes, which want the intellectual faculties of man. We must invariably examine the nervous system, not in an isolated matter, but with reference to the whole body; recollecting that it presents itself as the primary and most important structure regulating the general formation of the body, and, consequently, that the superiority of its rank is expressed, not merely by its individual formation, but also by that of the organism collectively. Consequently, even admitting, what is by no means the fact, that the brain is alike in man and apes, the latter must still rank below the former in a degree corresponding to the superiority of the general form of man. Neither must it be forgotten that this view of the result of the comparison of the simultaneous development of intellect and of the nervous structures is not by any means incompatible with the higher principle which forms the basis as well of the manifestations of the intellectual powers as of those of the nervous system: we have here to consider only what is perceptible by our senses, which, however, we must not confound with the principle itself. It is immaterial whether the object belong to the internal or external senses: in either case, it is still but an object of sense; but nothing can be more certain than that the ideas of sense, in the varied combinations of which our intellect consists, are, to the full, as much as corporeal subjects themselves, the mere perishable covering of the latent divine spark within.

"As to the observations which are intended to prove the permanence of this primary nervous power, independent of organic locality, they are totally inadequate for the purpose: an important injury is very far from amounting to absolute destruction: the permanence of sensorial manifestations, and of nervous action in general, after complete annihilation of the nervous system, would alone be decisive." (Vol. i. p. 275.)

*Organs of Sense.*

*Touch.*—In the vertebrated animals, as in those without vertebræ, the general surface is in many instances an universal organ of touch, while distinct parts devoted to that purpose are wanting: nay, in many species of the higher animals, this sense is either entirely wanting, or exists in a very imperfect degree. In Fishes and the Amphibia, the animal, in general, touches with the mouth alone; and in Birds the bill appears the only part where this sense is developed. Among the Mammalia, the palmata coincide in this respect with fishes; most animals having hoofs or claws with the amphibia, and bats with birds. It is remarkable that in the mole, shrew, pig, and elephant, the senses of touch and smell are combined in one organ, as in the inferior animals.

*Smell.*—M. Carus is of opinion that, as a true sense of smell can only be exercised in aeriform media, so it must necessarily be absent in those which do not respire air,—i. e. in fishes. It appears, however, that these animals are able to discover very slight changes of the fluid in which they are placed, (rapacious fishes, for instance, discover a carcase at a distance;) but this is attributed to a sense something mediate between taste and smell, and which our author calls *scent*. The general sense of smell, and that peculiar modification of it which is here called scent, have this in common with touch, that a certain degree of motion is essential to both kinds of sensation. There is little to remark with regard to smell in fishes, the amphibia, and birds. According to Scarpa, the sense is stronger in males of the last class than in females. In the mammalia, the organs acquire great additional development, and in many species have a distinct relation to the sexual system.

*Hearing.*—The organ of hearing in fishes is placed within the same cavity as the brain, and the little sac which we saw existed in the Mollusca is here accompanied by three semilunar canals, nearly resembling those of the human ear. In the Amphibia, the organs, originally within the cranium, are gradually more developed outwards, but in very different degrees. In Birds, the organs of hearing become more perfect; there is the rudiment of a cochlea, and the nerves are disposed essentially as in man. The Mammalia are distinguished, with regard to the organs of hearing, by the development of a true cochlea, by the multiplication of the auditory bones, and by an external ear. As to the peculiarities of these organs in man, it would appear that they are less calculated to receive delicate impressions than in the mammalia. Among the points of superiority in the latter, may be mentioned the size and mobility of the external ear in several species, the extent of the tympanum, the superior strength of the ossicula auditus, and the greater size of the auditory nerve, as compared to the size of the brain. But it is probable that the organization of the human ear is such as to adapt it for distinguishing varieties in sound to a greater extent than is possessed by any other animal. We thus perceive that in Fishes there is little more than the membranous labyrinth; in Amphibia and Birds, the tympanum is added, and the auditory bone of the fenestra ovalis is first found, whilst there is a commencement of an external auditory canal; and, in the crocodile and owl, even some traces of an external ear. In the Mammalia, the labyrinth is still more completely developed, and the tympanum still more completely separated from the articulation of the jaws, the bone of which, in birds and several amphibia, contribute to its formation.

*Sight.*—In Fishes and the Amphibia, the common integument is continued over the eye, and sometimes so little changed that the organ is completely hid by it, and must consequently be nearly insensible to light; the iris is but little moveable; and the ciliary processes are imperfectly developed. The size of the eye is considerable as compared to the brain, its position is generally lateral, and the orbit is for the most part incomplete. In the frog, the eyes project into the cavity of the mouth, and the animal has the power, by the action of a peculiar muscle, of concealing the eye by depressing it into the mouth. In the viper, the conjunctiva is so completely a prolongation of the common integument, that it is thrown off at the same time with it.

In Birds, the eye bears a remarkable proportion in point of size to the brain and to the head; a circumstance in which the organ here presents an analogy to that of insects. The conjunctiva and common integuments are entirely distinct in this class, and there are invariably three completely formed eyelids. The third eyelid, or membrana nictitans, is the most remarkable; it projects horizontally from the anterior angle of the eye, and is moved by a peculiar mechanism. It has attached to it a strong slender tendon, which passes round the eyeball, is braced away from the optic nerve by a small muscle, and ultimately terminates in a small pyramidal muscle, which, with the one above mentioned, is attached to the sclerotica, and serves to draw the membrana nictitans forwards. The sclerotica is very firm and elastic, consisting of three laminæ, between the outer and middle of which the osseous circle is inserted. This consists of from fifteen to seventeen laminæ of bone, forming in some cases a smooth circle, and in others a prominent cylinder. The cornea is generally very convex: in some birds (as the owl) a circle of muscular fibres has been discovered attached to its inner lamina, by which it becomes capable of being drawn inwards, nearly in the same manner as the fibres of the diaphragm depress its tendinous centre.

The peculiar characteristic of the eye in Birds is the pecten or marsupium. The optic nerve passes obliquely through the sclerotica, and expands to form the retina: immediately before the entrance of the nerve into the sclerotica, its laminated texture is in many places very conspicuous, when the eye has lain some time in alcohol: the central vessels pass into the eye between the laminæ, and they then unite into a blackish and nearly quadrangular membrane, arranged in beautiful folds, which, extending through the vitreous humor towards the capsule of the lens, form the marsupium.

In the Mammalia, the size of the eye is small relatively to the head, but it is more completely mobile than in the preceding classes: the lachrymal apparatus is more perfectly formed; and it ultimately presents itself in man as the most elevated organ of sense.

### *Electrical Organs.*

Intermediate between the organs of sense and those of motion may be ranked those which exert at will an influence, "partly nervous and partly electric," on external living objects. It is in fishes only that this power is manifested to any considerable extent,—viz. in the electric ray (Raya torpedo), the electric eel (Gymnotus electricus), and the Silurus electricus. Electric actions have likewise been observed in the Tetrodon electricus and the Trichiurus indicus. The structure of the organ in the three first is extremely like the common muscular fibre of fishes; it presents numerous strata, formed by tendinous partitions, filled with a whitish gelatinous fluid. Many nerves are distributed on these cells, and it is probable that the nervous power accumulates in these, and can be voluntarily discharged. In the higher classes of animals, the only things which can be at all compared with this phenomenon, are the electric light of the eyes and the electricity of the skin in cats. M. Carus is also of opinion that "in man the magnetic influence may be considered as a similar, though refined and modified appearance." We believe, however, that, on this side of the Channel, the magnetic influence is so much "refined" as to escape cognizance altogether.

A short account of the Organs of Motion terminates the volume; but these are not of a nature to interest our readers.

---

*A Manual of Surgical Anatomy, by* H. M. EDWARDS, D.M.P. *Translated, with Notes, by* WILLIAM COULSON, Demonstrator of Anatomy at the Medical School, Aldersgate-street, &c.—12mo. pp. 427. Simpkin and Marshall, London, 1827.

THE work before us is the production of Dr. MILNE EDWARDS, of Paris, who, it appears, had long entertained the design of publishing a work on Surgical Anatomy, and was actually engaged in the execution of it, when, in 1825, a treatise by M. VELPEAU* on the same subject made its appearance. But the plan of the treatise appearing to Dr. Edwards too minute for an elementary work, he completed his previous intention, and consequently published the Manual before us.

Surgical anatomy (in the true meaning of the term) embraces a wide and rich field for professional inquiry. It comprehends not only such a knowledge of the relative situation and action of parts as to enable a surgeon to perform operations with safety, and treat accidents judiciously, but it also points out the influence which the organisation, form, and situation of a part exercise on the development and treatment of those surgical diseases to which it is exposed. This latter branch of the subject has not been cultivated with the same success as the former, because those minute structures, upon a knowledge of which its successful cultivation depends, have not been generally studied, and are even yet by many but imperfectly understood. In Dr. Edwards' Manual we are glad to find considerable attention paid to the fasciæ in the different parts of the body, and their influence on the principal surgical diseases minutely pointed out.

Surgical anatomy has been called on the continent "anatomy of regions," because, for facilitating its study, the

* Traité d'Anatomie Chirurgicale; ou, Anatomie des Regions. 2 vols.—Paris, 1845.

division of the body into distinct regions is generally adopted. In this work the body is divided into seventeen of these; the anatomical descriptions of the parts in each region are given, and then the practical observations, to which these descriptions give rise, follow. Thus, after giving the anatomy of the liver, the author remarks:

"The relations which exist between the liver and surrounding parts are sufficient to explain the different passages by which the pus makes its escape externally in abscesses of the liver. In fact, if the matter be seated near the convex surface of the liver, adhesions may form between the parietes of the abscess and the diaphragm, and between this muscle and the lungs. Hence, by the process of ulceration, the pus may proceed into the cavity of the thorax, or even pass into the bronchi. If the abscess is situated near the anterior edge of the convex surface of the liver, adhesions may form between this part and the abdominal muscles, and the collection of matter may open externally into the right hypochondriac region, or even into the epigastric. Lastly, abscesses situated near the concave surface of the right lobe often empty themselves into the colon, and those placed near the inferior surface of the left lobe sometimes also open into the stomach." (P. 291.)

The following is an extract from the description of the anterior cervical region, which is well deserving the attention of our readers:

"Immediately under the skin, we find the superficial fascia, and between its two layers, the platysma myoides. This muscle is quadrilateral and very thin; it is attached inferiorly to the subcutaneous cellular tissue that covers the deltoid and pectoralis major; higher up, its fibres are directed obliquely upwards and inwards, and are lost above the lower jaw on the inferior part of the cheek. On the chin, the anterior part of the muscle is confounded with that of the opposite side, but it separates in descending, and thus leaves, on the median line, a triangular space, in which the two layers of the superficial fascia of the neck unite, and form a thick and resisting membrane. This fascia is continuous superiorly with that which covers the parotid gland: on the side of the neck it forms a dense layer, which, on the upper portion of the sterno-mastoid muscle, adheres strongly to the skin. Near the anterior edge of the trapezius, it loses its aponeurotic appearance; but it first sends off an elongation, which is attached to the temporal bone near the mastoid process, and which dips beneath the anterior edge of the sterno-mastoid muscle. Inferiorly, it is prolonged on the anterior surface of the sternum, under the form of subcutaneous cellular substance; and, on the upper edge of this bone, between the tendons of the sterno-mastoidei, it sends off an elongation, which proceeds backwards and downwards, and unites with another aponeurotic membrane. The superficial fascia of the neck is formed by the subcutaneous cellular tissue: in very fat individuals it is indistinct, whilst, in lean and anasarcous subjects, it has the appearance of an aponeurotic membrane; but it is chiefly when tumors exist in this region that it becomes strong and resisting: we, however, never perceive muscular fibres in it.

"The external jugular vein, which we have already seen descending from behind the parotid gland, upon the lateral part of the neck, continues its course immediately under the platysma myoides and the superficial fascia, towards the posterior edge of the inferior extremity of the sterno-mastoid muscle: it then descends vertically to reach the subclavian vein. During this course, it follows nearly the same direction as the fibres of the platysma myoides. When phlebotomy is performed here, it is, therefore, proper to make the opening more or less oblique in relation to these fibres.

"A vein descends from the lateral part of the face, runs under the superficial fascia on the fore part of the neck, and along the anterior edge of the sterno-hyoid muscle, and often acquires a considerable size near the bottom of the neck, and communicates with that of the opposite side by a pretty large branch. In the operation of bronchotomy, this anastomosing branch may be opened in dividing the integuments; and the blood that escapes might embarrass the surgeon, but it will not be productive of any bad consequence. In fact, this vessel, as well as its lateral branches, being situated immediately under the superficial fascia, and above another aponeurosis still deeper, which we shall presently notice, the blood will escape externally with greater ease than it can flow into the trachea.

"Under the superficial fascia of the neck, we find a second aponeurotic cellular membrane, which constitutes the deep fascia: it is fixed superiorly along the edge of the lower jaw, is continued laterally with the stylo-maxillary ligaments, and divides into two layers, to surround the sub-maxillary gland: it is attached to the projecting part of the thyroid cartilage, and again forms two layers. The anterior layer is extended upon the tendons of the sterno-mastoid muscles, and becomes continuous with the superficial fascia on the anterior edge of the sternum; the posterior proceeds to the posterior surface of the sternum, and is separated inferiorly from the anterior layer by a quantity of fatty cellular substance, which generally incloses some lymphatic glands. Upon the sides of the neck, this fascia degenerates into cellular tissue: its appearance also varies much, according to the individual; sometimes it is thin and cellular, at other times it is thick, and almost as distinct as the aponeuroses of muscles.

"One of the principal uses of the cellular layers which surround the inferior part of the neck, is to resist the pressure of the atmosphere in the moment of inspiring, and to prevent the trachea from being compressed by this force each time the thorax dilates. Thus when, in consequence of an ulcer, or other causes, these fasciæ, and the sterno-hyoidei and sterno-thyroidei muscles placed under them, are destroyed, the parts which at this point close the top of the thorax are not sufficient to resist the pressure of the atmosphere, and, when the chest dilates, they are forced forwards, and, by pressing on the trachea, produce a considerable difficulty in breathing.

"Some lymphatic ganglia, as we have said, are found above the edge of the sternum, in the adipose mass situated between the superficial and deep fasciæ. When these glands enlarge, a tumor arises, which may be mistaken for a morbid state of the thymus gland; but it does not produce the same degree of dyspnœa, the deep fascia preventing it from compressing the trachea. We also find lymphatic glands in other parts of the neck: some are placed above, and others below the fascia; and from these differences of situations arise great varieties, both in the characters and treatment of the tumors which they form. In fact, tumors situated between the integuments and cervical fascia are superficial, circumscribed, prominent, and moveable. For a long time, by pulling these tumors outwards, we can raise them sufficiently to be able to pass the fingers between their base and the neighbouring parts. They, however, form connexions at last with the fascia behind them, and cause either a thickening of that membrane and its adhesion to the adjoining parts, or else its absorption, in which case they sink among the deep parts to which they also become adherent. Therefore when, from their anatomical structure, extirpation is deemed advisable, the operation ought to be performed without delay, before adhesions form. The tumors which form under the cervical fascia are more frequent and more dangerous than those that form above that membrane. The resistance which this fascia offers to their development flattens them, and prevents the appearance of any external projection, before they have acquired a very considerable size. They, therefore, can only extend inwards, and sink between the muscles, vessels, and other deep parts: their extirpation is, consequently, attended with very great difficulty. This circumstance explains why those tumors occasion a difficulty in breathing, much more severe than their size would lead us to expect." (P. 89.)

Our limits will not allow us to give further extracts. The work contains a great deal of practical information, which cannot fail to be interesting to the surgical practitioner and the student. In another edition, we think plates, containing views of the parts concerned in the principal operations, might be given with advantage.

The translation is well executed, and Mr. Coulson has increased its value by the addition of notes containing information derived from the records of both English and German surgery.

*A Treatise on those Diseases which are either directly or indirectly connected with Indigestion; comprising a Commentary on the principal Ailments of Children.* By David Uwins, m.d.—London, 1827.

The work of Dr. Uwins is divided into three parts: the first relating to various general questions; the second to Diseases of Children, comprising desultory remarks on some of the complaints of Adults; the third to Indigestion.

The first division of the work is occupied with a tirade against all medical theories; in the course of which, however, the author shows himself to be one of the greatest theorists we have ever met with. All the peroration amounts simply to this,—that theory, if carried too far, is bad; that preconceived opinions and nosological distinctions must yield to facts; and that a man is more likely to learn the practice of physic at the bedside of the patient than in the closet: in all of which positions we entirely agree with Dr. Uwins.

The second chapter contains an anatomical and physiological account of the digestive apparatus, possessing nothing either new or attractive; and, in the third, we have a sketch of the differences of constitution observed in different individuals.

The second part opens with the consideration of "ailments principally of children, which originate in, or are connected with, faulty digestion." A child is described, much at length, as labouring under that combination of symptoms which is commonly implied in the term "remittent fever." In the course of the narrative, we are informed, among other things, "that an unhealthy excitement of *parts* is engendered by the torpor of the *whole*;" and that, while the arteries are doing too much, the lymphatics are doing too little. Now, the author is of opinion that the indications presented by this condition are most completely and satisfactorily supplied by digitalis. In short, the object of the entire section is to show that, while the "lymphatic organization requires a spur, the arterial impulse demands a bridle;" and foxglove is both the spur and the bridle: a piece of theory which the author very properly remarks, "may, or may not, be correct."

Hydrocephalic and convulsive affections are next spoken of in the same desultory manner. The only circumstance worthy of remark is, that in the former small doses of elaterium are recommended, and that the author, in some few cases where he believes effusion had taken place on the brain, met with "extraordinary success from tincture of cantharides, in doses of about four minims to a child of two years old, frequently repeated." In the convulsions of infants, he thinks bleeding is used too freely, and opium too much neglected. The beneficial influence of opium after bleeding is supposed to depend upon its "lessening the diameter of the capillaries," and thus preventing "that rush of blood again into them which is so apt to succeed to depletion of vessels." Indeed, it is remarkable throughout that Dr. Uwins, who sets off by condemning theory, has an hypothesis for every thing.

Under the head of Worms, which comes next, there is not a single remark worthy of being quoted; and, in the sections which follow on various forms of eruptions, the only circumstance with which we shall trouble our readers, is the author's statement that he uses largely and successfully in "skin disorders" the following prescription. It is unchemical, but we believe that chemical principles have very little to do with curative effects.

℞. Ammoniæ Subcarb. ℈ss.; Magnesiæ Sulphat. ʒj.; Mist. Camphoræ ℥jss. Fiat haustus.

In Hooping-cough, the combination of "cochineal with salt of tartar" is highly praised, and the effects of Roche's embrocation are said to be sometimes "miraculous."

A few remarks on Croup, in which the common treatment of bleeding, calomel, and antimony is recommended, terminate the chapter.

The author next proceeds to speak of Consumption, under which term are included simple abscess of the lungs, malignant tumors ("soft cancer,") schirrus, and syphilis, as well as true tubercular phthisis. In this blending together of diseases entirely different in their nature, the only circumstance which arrests our attention is the assertion that the author has "frequently witnessed" consumption dependent upon schirrus of the lungs. This we apprehend to be a mistake: schirrous degeneration of the lungs is a very rare occurrence, and we suspect either that he uses the term vaguely, or else that he has not sufficiently distinguished between true schirrus and other indurated conditions of the lung, bearing more or less resemblance to it. Attention to diet, exercise, and climate are enjoined, much in the same way as in every other work of the kind published during the last twenty years.

Asthma is justly said to be greatly modified by the state of the digestive organs, and the views of our author on the subject may be conveyed in very few words, and those his own:—"Of medicinals for stomach asthma, the two main ones may be set down as soda and rust of iron;" and again—"During the violence of the paroxysm, a liberal use of those medicinals which go under the name of antispasmodics may be safely had recourse to."

Although, in some of his sections, we think our author has discussed matters but very remotely connected with the avowed object of the work, yet this remark does not apply to Affections of the Heart, which come next to be considered. Dr. Uwins alludes to the difficulty of distinguishing functional from organic diseases, and mentions some cases in which patients have entirely recovered, although the evidence of change of structure appeared *a priori* to be very strong. He relates the case of a friend of his, whose brother died of organic disease of the heart, and who for a long time laboured under symptoms which led to the apprehension of his having become similarly affected. He took many remedies without avail, and at length was permanently relieved by the spontaneous supervention of a bilious diarrhœa. Dr. Uwins is anxious to show that it was not the mere discharge of bile, however, which produced this effect, but some occult change, concerning which the author himself does not appear to entertain very precise ideas. "It is (says he) a change in the sentient organization, eluding the sight and almost the conception, that regulates often these sympathetic and *mimositic* maladies, and which are occasionally referred to a matter retained, while that something, the expulsion of which is consentaneous with the cure, seems to be manufactured, as it were, in the moment, and is merely the manifestation or effect of the required change which has set all to rights."

In the immediate attacks of "heart affections," Dr. Uwins thinks the principal difficulty consists in determining whether they depend upon accumulation of blood in quantity greater than the organ is capable of propelling, or whether the heart suffers collapse from a want of the requisite supply of nervous energy. It is certainly quite conceivable that either of these conditions may cause the heart's action to be interrupted or to cease, but we are sorry that we cannot give from the work before us any satisfactory method of distinguishing between them. Even in those cases where bleeding has been required, and *a fortiori* in those where it is not, stimuli are regarded as necessary; and of these, ammonia is held to be the best.

A succession of short chapters, or sections, follow, on Apoplexy and other subjects; but they are so wholly destitute of interest, that we cannot fix on a single novelty to present to our readers. Passing these by, therefore, without farther remark, we proceed to consider a very important question—the means of distinguishing between sympathetic and organic diseases. The pulse is said to be one of the best means of forming this diagnosis. Sudden variations in the frequency of the pulse, depending upon the mental feelings of the patient, are characteristic of merely nervous affections; while the quickness of pulse which is permanent, and little, or not at all, influenced by the moral impressions of the patient, is generally connected with structural disease: in other words, the former, proceeding from a transient cause, gives rise to a transient effect; while the latter, resulting from a permanent cause, produces an effect which is also permanent. The pulse, too, is stated to be harder when the blood-vessels are engaged in the disease, than when this is limited to the nerves. But Dr. Uwins does not place much confidence in those minute shades of difference in the character of the arterial pulsations, which have been described, and "some (he remarks) may probably affect to have, or cheat themselves into the supposition of having, more delicacy of perception than is positively the case; just as some would profess to feel or fancy they felt more pleasure in musical sounds than they actually do, merely because the want of the pleasure might be thought to imply a general deficiency of sentiment or taste." There is some justice in this remark.

The state of the spirits, also, assists in the decision: they who fear most having generally least cause to fear; and the actual supervention of an organic disease sometimes producing a certain degree of firmness and fearlessness of mind. The countenance, too, is characteristic of the presence or absence of organic change; and here, likewise, permanency is connected with disease of structure, mutability with disorder of function. To give any correct idea of varieties of expression by words must at any time be difficult, and, with the vague language of our author, the attempt would be hopeless.

The supervention of hectic is mentioned as indicative of disorganisation, but it is too obvious to require that we should dwell upon it.

The presence of much flatus is looked upon as favorable: "for the most part, (says our author,) where there is much flatus, there is little of formidable disease." This is unquestionably true with regard to those affections commonly understood by the term nervous; but in other cases, it is just the reverse. Thus a flatulent and tympanytic state of the abdomen, in the advanced stage of fever, is indicative of the presence, not the absence, of change of structure.

Dr. Uwins thinks that seeing and feeling are of more use to the physician than hearing, and speaks rather more disrespectfully than some will like of the stethoscope, many of the boasted discoveries of which he holds (and we agree with him) are not made "till you have pretty well made them out by other means."

We have now arrived at the commencement of the subject which constitutes the proper business of the volume, and at the end of our analysis. The third part treats of digestion, "in the abstract and popularly," and consists of a running commentary on the works of Dr. Phillip, Dr. Paris, and Dr. Johnson. It is our object to convey to our readers as faithful an idea as possible of any thing in the works which we review that may be peculiar, either in matter or manner; and, when we find neither, we think it an act of kindness, both to them and the author, to shut the volume.

### COLLECTANEA.

Floriferis ut apes in saltibus omnia libant,
Omnia nos, itidem, depascimur aurea dicta.

